# Development of a smartphone screening test for preclinical Alzheimer’s disease and validation across the dementia continuum

**DOI:** 10.1186/s12883-024-03609-z

**Published:** 2024-04-16

**Authors:** Jane Alty, Lynette R. Goldberg, Eddy Roccati, Katherine Lawler, Quan Bai, Guan Huang, Aidan D Bindoff, Renjie Li, Xinyi Wang, Rebecca J. St George, Kaylee Rudd, Larissa Bartlett, Jessica M. Collins, Mimieveshiofuo Aiyede, Nadeeshani Fernando, Anju Bhagwat, Julia Giffard, Katharine Salmon, Scott McDonald, Anna E. King, James C. Vickers

**Affiliations:** 1 Wicking Dementia Research and Education Centre, University of Tasmania, Liverpool Street, Hobart, TAS 7001 Australia; 2 School of Medicine, University of Tasmania, Hobart, TAS 7001 Australia; 3 School of Allied Health, Human Services and Sport, La Trobe University, Melbourne, VIC 3086 Australia; 4 School of Information and Communication Technology, University of Tasmania, Hobart, TAS 7005 Australia; 5 Royal Hobart Hospital, Hobart, TAS 7001 Australia; 6 School of Psychological Sciences, University of Tasmania, Hobart, TAS 7005 Australia

**Keywords:** Alzheimer’s disease, Dementia, TapTalk, Finger-tapping, Diadochokinesis, Computer vision, Kinematics, Digital biomarker, Artificial intelligence, Multimodal, TAS Test

## Abstract

**Background:**

Dementia prevalence is predicted to triple to 152 million globally by 2050. Alzheimer’s disease (AD) constitutes 70% of cases. There is an urgent need to identify individuals with preclinical AD, a 10–20-year period of progressive brain pathology without noticeable cognitive symptoms, for targeted risk reduction. Current tests of AD pathology are either too invasive, specialised or expensive for population-level assessments. Cognitive tests are normal in preclinical AD. Emerging evidence demonstrates that movement analysis is sensitive to AD across the disease continuum, including preclinical AD. Our new smartphone test, TapTalk, combines analysis of hand and speech-like movements to detect AD risk. This study aims to [1] determine which combinations of hand-speech movement data most accurately predict preclinical AD [2], determine usability, reliability, and validity of TapTalk in cognitively asymptomatic older adults and [3], prospectively validate TapTalk in older adults who have cognitive symptoms against cognitive tests and clinical diagnoses of Mild Cognitive Impairment and AD dementia.

**Methods:**

**Aim 1** will be addressed in a cross-sectional study of at least 500 cognitively asymptomatic older adults who will complete computerised tests comprising measures of hand motor control (finger tapping) and oro-motor control (syllabic diadochokinesis). So far, 1382 adults, mean (SD) age 66.20 (7.65) years, range 50–92 (72.07% female) have been recruited. Motor measures will be compared to a blood-based AD biomarker, phosphorylated tau 181 to develop an algorithm that classifies preclinical AD risk. **Aim 2** comprises three sub-studies in cognitively asymptomatic adults: (i) a cross-sectional study of 30–40 adults to determine the validity of data collection from different types of smartphones, (ii) a prospective cohort study of 50–100 adults ≥ 50 years old to determine usability and test-retest reliability, and (iii) a prospective cohort study of ~1,000 adults ≥ 50 years old to validate against cognitive measures. **Aim 3** will be addressed in a cross-sectional study of ~200 participants with cognitive symptoms to validate TapTalk against Montreal Cognitive Assessment and interdisciplinary consensus diagnosis.

**Discussion:**

This study will establish the precision of TapTalk to identify preclinical AD and estimate risk of cognitive decline. If accurate, this innovative smartphone app will enable low-cost, accessible screening of individuals for AD risk. This will have wide applications in public health initiatives and clinical trials.

**Trial registration:**

ClinicalTrials.gov identifier: NCT06114914, 29 October 2023. Retrospectively registered.

**Supplementary Information:**

The online version contains supplementary material available at 10.1186/s12883-024-03609-z.

## Background

Dementia has been described by the Lancet 2017 Commission as *“the greatest global challenge for health”* [1]. There are huge personal costs for people living with dementia and their families and huge economic costs for social and healthcare systems. With people living longer than ever before, and age the main non-modifiable risk factor for dementia, the prevalence is predicted to triple to 152 million worldwide by 2050 [1]. This is particularly pertinent in Australia with its rapidly ageing population and the number of Australians diagnosed with dementia doubling in the last decade to over 400 000 [2]. The key strategy to reduce prevalence is prevention [3, 4] as evidence suggests that about 40% of dementia cases are attributable to modifiable risk factors such as physical inactivity, hypertension and smoking [5]. Strategies to modify these risk factors, and clinical trials of new neuroprotective drugs, are likely to have their greatest impact if they target high-risk individuals at the earliest stages of disease [4, 6]. However, there are currently no population-level screening tests to detect the underlying brain pathology of dementia in the earliest stages. This lack of effective screening tools is a major barrier to reducing the prevalence of dementia. The ability to detect early ‘silent’ pathology would revolutionise the effectiveness of prevention strategies from a blunted ‘one size fits all’ approach to early precision targeting of high-risk individuals before cognitive symptoms emerge and before the brain is irreparably damaged.

The most common cause of dementia is Alzheimer’s disease (AD), accounting for 70% of cases [7]. The pathology of AD includes accumulation of abnormal proteins such as amyloid-beta (Aβ) and phosphorylated tau (p-tau) in the brain, together with neuronal degeneration, glial activation, and neuroinflammation [8]. This pathology covertly progresses for about 10–20 years before symptoms (such as poor memory) and signs (such as low scores on cognitive tests) of dementia are evident [5, 8]. Across the disease continuum, there are three key stages of AD pathology: preclinical AD (before any cognitive symptoms or signs emerge), Mild Cognitive Impairment (MCI), which is also called “prodromal AD” (where cognitive decline has occurred but not to the stage to impair functional abilities), and AD dementia. Cognitive tests commonly used clinically, such as the Montreal Cognitive Assessment (MoCA), and the Mini-Mental State Examination (MMSE), lack sensitivity until the MCI stage, when AD pathology has progressed for around 10 years [9, 10]. Specialist biomarkers such as cerebrospinal fluid (CSF) tests and positron emission tomography (PET) brain scans can detect AD pathological changes across the continuum, including in preclinical AD, but are rarely used clinically as they are too expensive, inaccessible, or invasive and would not be suitable for population-level screening [11]. Even with the recent emergence of blood-based biomarkers such as p-tau 181 [12] (that will be used individually, or in combination with others, as the biomarker-defined measure of AD pathology in this study), it remains unclear how accessible, or costly, these will be for widespread use [13, 14]. Thus, there remains an urgent, unmet need for low-cost, population-level tests to detect AD pathology across the continuum, especially in the earliest stages.

For over a decade, it has been recognised that movements of the human body gradually change across the continuum, and this begins in the preclinical AD stage [15]. In particular, there is a substantial body of research assessing how gait (walking) patterns change. Several studies using gait mats and/or wearable movement sensors to precisely analyse gait patterns have shown that it is possible to detect risk of AD pathology in the preclinical stages [16]. For example, in a cohort study of more than 3,500 older adults with normal cognition at baseline, the speed of walking slowed down 7 years before dementia onset [17]. However, gait analysis has several limitations as a population-level test including falls risk, variation with obesity and the need for specialist movement analysis equipment.

An emerging body of new research demonstrates that analysis of hand movements is also sensitive to preclinical AD pathology and may be a more accessible population-level test [18–20]. For example, in a sample of about 70 older adults, Mollica et al. found that speed and variability of repetitive key-tapping hand movements progressively declined in preclinical AD and AD dementia, and that finger tapping variability (a measure of the irregularity of rhythm) positively correlated with a fluid biomarker of AD pathology (CSF Aβ levels) [20]. Our own research, in > 1,200 cognitively asymptomatic older adults, found that key-tapping hand movements were slower, less rhythmic and had longer key-dwell times in those with lower scores on episodic memory tests (a proxy measure of preclinical AD) [19]. Other studies found that hand reaction times correlated with CSF Aβ levels and worsened across the AD continuum [15, 21, 22]. A recent scoping review of 60 studies, comprising 41,800 participants, examined the associations between a wide range of upper limb movements and cognitive impairment and found that, generally, slower and less rhythmic movements associated with cognitive decline, but only 5 studies (with 310 participants in total) examined finger-to-thumb tapping [18].

There is also evidence that analysis of oral movements may be sensitive to AD. Syllabic diadochokinesis (DDK) is a clinical test requiring adults to say ‘pa’, ‘ta’ and ‘ka’ repetitively, in isolation and in combination, so speech pathologists and other clinicians can evaluate the speed, rhythm, accuracy and coordination of lip, tongue, and palate movements respectively [23]. Clinically, the DDK test is used to detect types of motor speech disorders: (i) abnormalities of articulation secondary to weakness or incoordination of the muscles required for speech (dysarthria), and (ii) speech production difficulties due to sequencing or programming of muscles in the absence of weakness or incoordination (apraxia) [24]. Two types of DDK tasks are commonly used: Alternating Motion Rate refers to the rapid repetition of single syllables such as ‘pa’ or ‘ta’ or ‘ka’; Sequential Motion Rate refers to the rapid repetition of syllables in sequence, ‘pa-ta-ka.’ Both tasks are valid and sensitive motor tests and used in the differential diagnosis of adults with a variety of neurological disorders [25, 26]. Alternating Motion Rates are less affected by linguistic factors. However, Sequential Motion Rates are more complex as they require rapid and successive programming of an unfamiliar non-word motor sequence [24].

Recent research suggests that DDK is a biomarker of cognitive decline, with age-related changes in sensory, motor and language systems impairing cognitive processing and task performance [23, 27, 28]. The DDK test is valuable in diagnosing and monitoring functional decline in progressive neurological disorders, such as dementia, but syllabic DDK has never been investigated in preclinical AD and only once before been assessed precisely using computer analysis [29]. Typically, DDK tasks are audio-recorded, then played back for manual and subjective analysis of rate, rhythm, and accuracy by a speech pathologist. In this project, we will develop an online automated version of the clinical test and apply computer technologies to increase efficiency and accuracy of analysis.

The neural basis for motor manifestations in the preclinical stages of AD, and across the disease continuum, remain uncertain. Most motor studies have evaluated gait dysfunction and generally these have found associations between slower walking speeds with higher amyloid burden [30], smaller hippocampal volume (an area of the brain critical for memory function) [31] and prefrontal deactivation [32]. In terms of hand movements, a recent study found people with MCI and AD dementia had slower and less regular key-tapping hand motor performance with the severity of impairment associated with smaller hippocampal volumes but not with global Aβ deposition [33]. A functional MRI study of more than 600 participants also found evidence that the earliest stages of AD are associated with alterations (less network segregation) in the hand and mouth motor areas of the brain as well as the cognitive association areas [34]. Notably, the AD-related network alterations were independent of amyloid pathology, and distinct from aging-related functional network alternations that usually spare sensory-motor systems relative to association systems [35].

Thus, we propose that looking beyond the current definition of dementia – a clinical syndrome of cognitive decline – and instead investigating AD via movement analysis will facilitate the development of a new test that can detect AD across the continuum, including the preclinical phase. Specifically, we plan to combine analysis of repetitive hand movements (finger-to-thumb tapping) with analysis of speech-like movements (through the DDK test) to provide a more sensitive and inclusive means for detecting AD pathology. This ‘multimodal’ approach will use digital video data for finger tapping analysis combined with audio data for DDK data and is expected to improve the accuracy of the test compared to using a single type of test; a scoping review of 46 studies and 11,750 participants found that, applying a multimodal approach improves the sensitivity and specificity of tests to detect AD and other neurodegenerative disorders [36]. In further support of this proposal, it is noteworthy that the hand and lips-tongue-palate areas in the cerebral cortex share a close topographical relationship, suggesting shared movement pathways [37]. We also recognise from our previous research (see TAS Test Project) [38] that co-morbidities mean that some people cannot finger-tap (for example, limited by pain or paralysis) and that analysing a wider range of hand movements, and two types of movement test (speech-like and hand movements) will be more inclusive. However, repetitive hand and speech-like movements have never been specifically investigated together as a test for the preclinical stage of AD, or indeed for any stage of the AD continuum.

We will use cutting-edge Artificial Intelligence (AI) - based technologies, building on our previous research, to automatically analyse hand and speech-like movement from digital video and audio recordings respectively, and to ‘learn’ abnormal patterns of data that are associated with biomarker-defined AD pathology [39–44]. These advanced computer science techniques provide a precise, automated and efficient method of analysis. We previously developed machine learning techniques for measuring finger tapping using laptops and research cohorts i.e. people who do not have cognitive symptoms and found this approach to be feasible and sensitive to cognitive performance [19, 38, 42, 43]. The proposed method of TapTalk is non-invasive and, as the tests are movement-based rather than language-based, we have purposefully considered that they are accessible to culturally and linguistically diverse communities, First Nations peoples, and those with low literacy skills.

## Methods

### Aims and hypothesis

The overall aim of this project is to develop TapTalk – a smartphone test that detects risk of AD pathology and is usable, reliable and validated against blood-biomarkers of AD pathology, cognitive screening tests and clinical diagnosis. We will address the hypothesis: “*Hand-speech movement patterns will detect the risk of Alzheimer’s disease pathology in research and clinical cohorts of older adults*”, where research cohorts have normal cognition and no cognitive symptoms, and clinical cohorts have cognitive symptoms.

Our interdisciplinary team of clinician-researchers (Neurologist, Speech Pathologist, Physiotherapist, General Medicine Physicians, General Practitioner (GP), Neuropsychologist), computer scientists (specialising in Artificial Intelligence and machine learning), data analysts, and neuroscience researchers (specialising in ageing, dementia and blood-based biomarkers) will work together to address the hypothesis through three studies, with each study addressing one of the following aims:

- **Aim 1.** Determine which combinations of hand-speech movement data most accurately predict preclinical AD.
- **Aim 2.** Develop smartphone capability for TapTalk and determine usability, reliability and validity.
- **Aim 3.** Prospectively validate TapTalk in people who have cognitive symptoms against gold-standard consensus clinical diagnoses of MCI and AD dementia, with comparison to other screening tools commonly used in clinical settings.

### AIM 1

#### Design

**Fig. 1:**
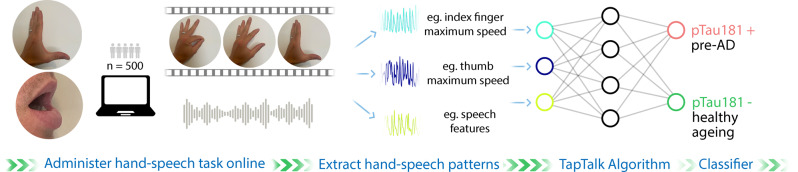
Cross-sectional study (n = 500) to identify hand-speech motor biomarker classifier

We will undertake a cross-sectional single site study. Overview of the methods is presented in Fig. 1.

#### Participants

We aimed to recruit at least 500 eligible participants (see below for inclusion and exclusion criteria) with normal cognition from the ISLAND Project. As of March 2024, we have recruited 1382 adults, with mean (SD) age 66.20 (7.65) years, range 50–92 years, and 72.07% female. The ISLAND project was launched in October 2019 at the University of Tasmania as a 10-year prospective cohort study of Tasmanians ≥ 50 years old with nested interventions to reduce dementia risk [45, 46]. To date, > 14,000 people have been recruited to the ISLAND Project; 8,500 research participants (mean age 63 years) have provided detailed demographic and health data, 3,000 have completed the online validated Cambridge Neuropsychological Test Automated Battery (CANTAB) cognitive tests [47], 1,800 have completed online motor-cognitive tests (TAS Test) [38] and more than 1,500 have provided blood samples. Paired Associates Learning (PAL) scores distinguish between older adults with MCI and those who are cognitively healthy with a sensitivity/specificity of 0.83/0.82 [48]. The online ISLAND Project surveys are repeated annually with cognitive performance and blood-based biomarkers collected every second year.

##### Inclusion criteria

Adults ≥ 50 years old who are (i) participants in the ISLAND Project [45] (ii) have provided a blood sample for analysis of blood-based biomarkers, (iii) have *normal* cognition defined as not ≥ 2 SD above the mean total errors (adjusted for age) on the CANTAB PAL tests, and (iv) have no history of persistent cognitive symptoms reported on ISLAND Project questionnaires (all participants are asked each year: *Have you noticed a substantial change in your memory and mental function in recent years*? [YES/NO], *Have you been told by a doctor that you have dementia?* [YES/NO], *Have you been told by your doctor that you have a memory impairment but they were uncertain if you have dementia?* [YES/NO], *Have you discussed concerns about your memory and mental function with your doctor or other health professional?* [YES/NO]).

##### Exclusion criterion

Adults < 50 years old or those who have impaired cognition, defined by a diagnosis of dementia or MCI or a validated cut-off score ≥ 2 SD above the mean total errors, adjusted for age, on the CANTAB PAL tests or reporting ‘YES’ on any of the ISLAND annual questions ‘*Have you noticed a substantial change in your memory and mental function in recent years?*’ *Have you been told by a doctor that you have dementia?* [YES/NO], *Have you been told by your doctor that you have a memory impairment, but they were uncertain if you have dementia?* [YES/NO], *Have you discussed concerns about your memory and mental function with your doctor or other health professional?* [YES/NO]).

As part of the ISLAND Project, we will measure blood levels of a range of proteins known to be associated with AD pathology, including p-tau 181 [45, 49], glial fibrillary acidic protein (GFAP) and neurofilament light (NFL) [45, 49]. Although minimally invasive, the practicalities and cost of accessing the specialist analytic equipment limit wide accessibility. We will use ultrasensitive Simoa® immunoassays measured using the SR-X™ platform from Quanterix™ for blood biomarker analysis in a dedicated biobank with − 80 °C freezers for sample storage.

Based on the latest scientific literature, and recognising emerging work around variability we will either define cut-off ranges for biomarker levels that are indicative of risk for AD dementia or examine associations across continuous measures [50]. This ‘AD biomarker’ in the TapTalk project is likely to be based on one or more blood tests for validated proteins such as p-tau181, GFAP and NFL [12, 51, 52]. The field of AD blood biomarkers is developing rapidly [14], and we anticipate further pathological markers will become available during the life of this project; we will assess such new markers in the samples collected from study participants.

### Setting

Most participants will complete the hand and speech-like movement tests online remotely either at home, or another setting of their choosing, using their own laptop or desktop computer. If they cannot access a computer with a computer camera and microphone, they may attend one of the University of Tasmania research sites to complete the tests on a university laptop. The TAS Test software is an online ‘self-test’ program that guides the participant through a series of screens to collect a range of motor, cognitive and speech tests [38]. It has already successfully collected finger tapping and hand movement data online (as well as various cognitive tests) from ∼1,800 ISLAND Project participants at home and ∼400 participants at University of Tasmania research settings [19, 42]. For the TapTalk project, the TAS Test software will be adapted to collect new finger tapping test data and include an online version of DDK tasks to collect speech-like data.

**Fig. 2:**
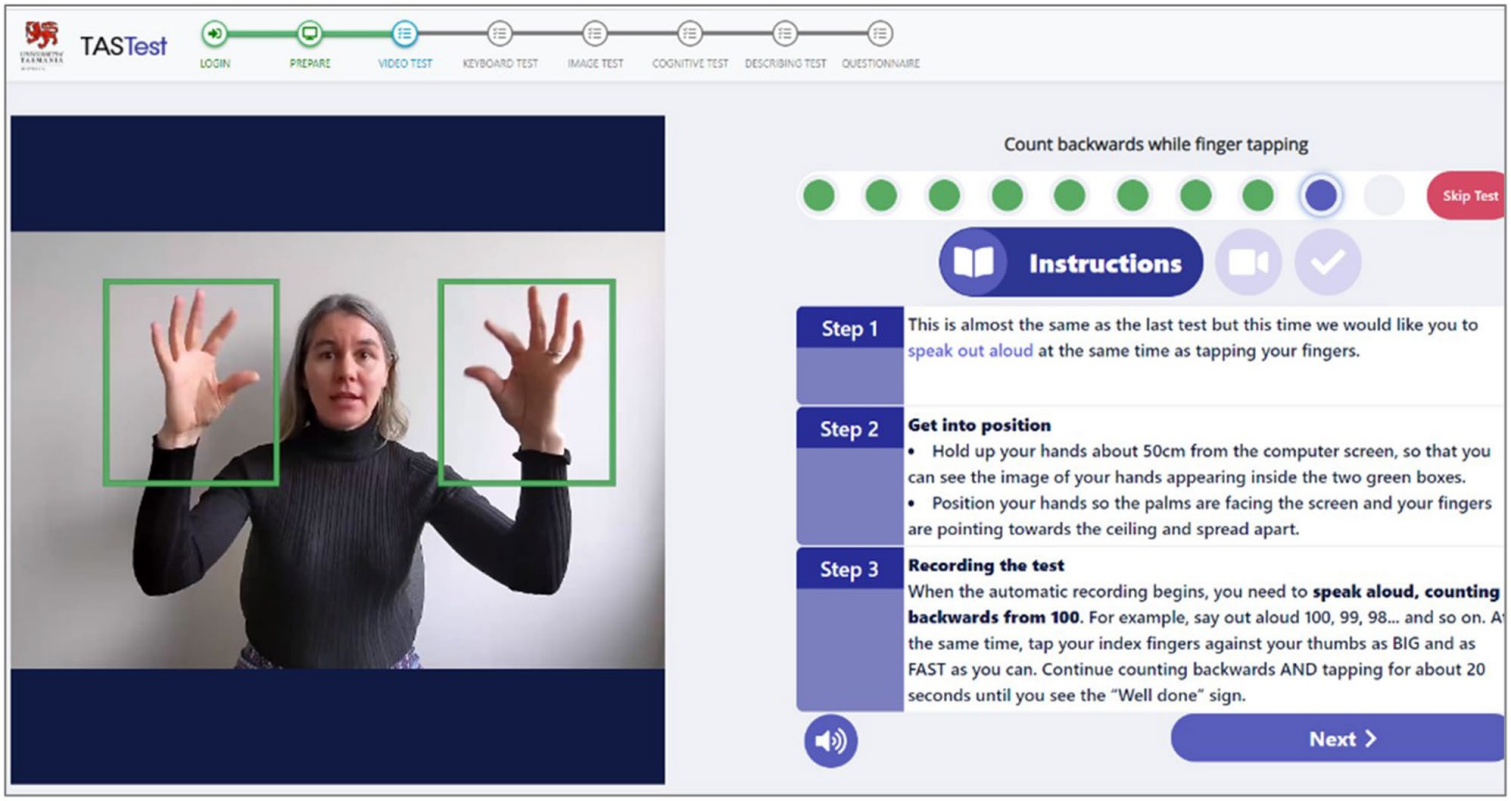
Example of an instruction screen in TAS Test for a finger tapping task [38]. The video instructions are on the left, the text instructions on the right, with the audio icon underneath (so participants can listen to the instructions if preferred). When the participant is ready to attempt the task, they press the ‘Next’ button to move on to the ‘recording’ screen. The green circles above the written instructions show the participant their progress through the various tasks

The full protocol of TAS Test is described in Alty et al.; [38] in brief, when participants log into the TAS Test software, and provide consent online to take part, they are shown an introductory video and general instruction screens. Next, they are asked for permission for the software to switch on their computer camera and microphone. Each individual test (e.g., ‘right hand finger tapping at comfortable pace’ test) within TAS Test has an ‘instruction’ screen followed by a ‘recording’ screen, followed by a “*well done, you have completed the test*” message. To move to the next screen, the participant clicks the ‘next’ button which means the tests are self-paced and participants can take a break at any time. They are shown their progress through TAS Test by green dots on a ‘progress bar’ at the top of the screen; see Fig. 2.

On each instruction screen, participants will be shown a 5–10 s looped video that demonstrates the hand, or speech-like movement that is required for the task. There are text instructions next to the video and participants may click on an ‘audio’ icon if they would like to hear spoken instructions (the text instructions are read out aloud). When participants are performing the hand or speech-like movements on the ‘recording’ screen, the computer video camera will record the hand movement data and the computer microphone will record the audio data respectively.

### Hand movement data collection

For each of the hand movement tests, the participant is asked to hold their hands up about 50 cm in front of the computer camera and adjust their position until they can see an image of their hands on the screen fitting completely inside green ‘data collection’ boxes. This careful positioning of the hands at the start of each test assures that the camera can record the movement patterns accurately. Computer-user interface technologies are embedded into the TAS Test software that respond to the positioning of the participant’s hands and give them on screen text prompts (e.g., “your right hand is correctly positioned”, “move your left hand closer to the screen”, or “well done your hands are in the correct position”) to prompt adjustments in the hand positioning as required. These technologies have been purposefully included to allow TAS Test to collect robust data at home, or any remote setting, without any in-person researcher assistance being required [42].

The hand movement tests comprise tests of repetitive finger tapping (index finger tapping against thumb) tests, and of repetitive ‘sequential’ finger tapping (index, middle then ring finger each in turn tapping against the thumb, then index, middle, ring etc.). The sequential finger tapping tests are a commonly used clinical test of motor function chosen in this study for direct comparison with the Sequential Motion Rate analysis in the DDK test. Each test is about 10 s duration and completed in a fixed order as follows: (i) at a comfortable pace: right hand finger tapping, left hand finger tapping, right hand sequential finger tapping, left hand sequential finger tapping; (ii) at a maximal pace ‘as big and fast as you can’: right hand finger tapping, left hand finger tapping, right hand sequential finger tapping, left hand sequential finger tapping; (iii) at maximal pace: both hands finger tapping together in phase, both hands finger tapping together out of phase, both hands finger tapping together in phase with a cognitive task, both hands finger tapping together out of phase with a cognitive task.

‘In phase’ means that both hands move simultaneously through the finger tapping cycle, whereas ‘out of phase’ means that each hand moves through the finger tapping cycle in the opposite direction to the other hand (i.e., 180 degrees out of phase so one hand has the finger and thumb opposed while the other hand has the finger and thumb spread apart). The cognitive task will be counting backwards aloud from 100 and constitutes a ‘dual motor-cognitive test’ when performed at the same time as finger tapping [53, 54]. Each hand will therefore be tested separately, and together, at a comfortable pace and at maximal pace, allowing for a number of finger-tapping measures to be calculated including, but not limited to, frequency, rhythm, inaccuracies (i.e. the wrong digit tapped on the sequential tests), ‘motor reserve’, calculated as a ratio (the maximal pace parameter divided by comfortable pace parameters), bimanual ‘motor cost’, calculated as the finger tapping parameters of one hand divided by the finger tapping parameters of same hand when both hands tapped together and the ‘dual cognitive-motor cost’, calculated as the finger tapping parameters divided by the finger tapping parameters during the counting backwards aloud from 100 task.

We will shorten this finger tapping protocol to around three to five tasks, aiming for a test duration of 2 to 3 min, in the smartphone version of TapTalk that will be used in Aim 2 and Aim 3. The choice of which tasks will comprise the shortened protocol will be based on the findings from Aim 1. We have purposefully kept a wide array of tests in Study 1 to clarify which tests combine best together, and with the DDK data, to best discriminate biomarker-defined AD pathology.

### Speech-like data collection

It is unclear which tests are most sensitive in preclinical AD. We will replicate the clinical DDK [23, 27] test but under both comfortable and maximal speed conditions. A participant will be instructed to say “pa, p.a., pa…” repetitively for 10 s at a comfortable pace, then the participant will be instructed similarly for “ta, ta, ta…”, “ka, ka, ka…” and “pa-ta-ka, pa-ta-ka…” all at a comfortable pace. After this, they will be instructed, to repeat each test in turn but this time at maximal pace. These deceptively simple tests assess the motor function of the lips (p.a.), tongue (ta) and palate (ka) respectively, allowing us to calculate the speed, rhythm, accuracy, and motor reserve (‘maximal pace’ measures divided by ‘comfortable pace’ measures) for each syllable.

**Fig. 3:**
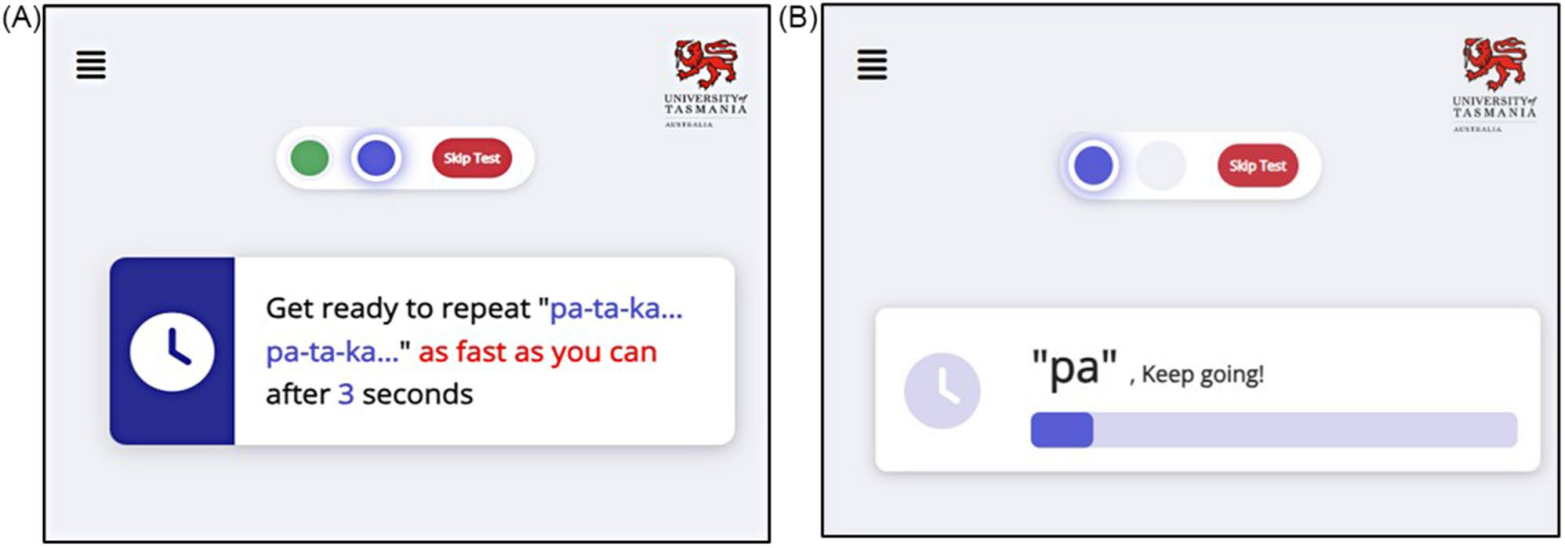
Speech-like test protocol: (**A**) a ‘get ready’ screen appears for 5 s immediately after the instruction screen (not shown); (**B**) the recording screen includes brief prompts such as “pa” and a visual count down for 10 s to encourage participants to keep repeating the speech-like sounds for the full period of recording

Similar to the format of the hand movement tests, there will be an ‘instruction’ screen and a ‘recording’ screen followed by a “well done” screen. The difference compared to the hand movement tests, in terms of the software, is that the recording screen does not show a video image of the participant as they perform the task; see Fig. 3. At the end of the protocol, the participant will be asked to complete a questionnaire on their experience of the tests and suggestions for improvement.

### Extracting hand-speech movement data

We will use deep neural networks and other machine learning methods to develop and train two computer programs that will automatically detect, respectively, hand key points (e.g. finger and thumb tips) in the video data [43], and syllable features in audio recording (Fig. 1). A series of movement features will be extracted in a fixed window period, by (1) experts in hand movement (JA, KL, RSG) and speech-like movements (LG), and (2) deep-learning approach applied directly on processed video data, to analyse their associations with biomarker-defined measures of AD pathology. The processed video data can be the hand-only cropped video or displacement (between fingertip and thumb-tip) over time data extracted from the raw video. Thus, two approaches, expert guided and AI-based ‘deep-learning’, will extract discriminative hand-speech movement features as input data for the multivariable model. The main outcome will be a validated personal computer (laptop and desktop) version of the TapTalk that discriminates preclinical AD from normal ageing in cognitively healthy adults ≥ 50 years old with defined sensitivity and specificity.

### Statistical analysis

We will use data from 350 of a sample of 500 participants to develop multivariable models that classify data according to risk of preclinical AD pathology. Data from the remaining 150 will determine the externally validated sensitivity/specificity of candidate models in detecting preclinical AD. Hand-speech movement data will be normalised. Participants with blood biomarker levels in the preclinical AD range (e.g., p-tau 181, or a combination of blood biomarkers) will be classified as ‘AD positive’. We will use penalised logistic regression to measure the associations between movement patterns and AD positivity. Covariates may include age, gender, apolipoprotein E ([APOE] a risk gene for AD analysed through blood sampling as part of the ISLAND Project), years of education, and handedness. As a secondary, more agnostic approach, we will use deep learning methods to discover features in movement and speech-like data that map to AD positivity.

We will use cross-validation to avoid overfitting by selecting penalty terms (type and amount of shrinkage applied to coefficients) which optimise the bias-variance trade-off. Bootstrap procedures will be used to estimate model uncertainty. We will externally validate models using data from the remaining 150 participants. Receiver Operating Characteristic (ROC) curves will be plotted against the positive AD cut-off to assess the sensitivity/specificity of movement and speech-like models to discriminate the preclinical AD stage from normal ageing. The model with the highest area under the ROC curve (AUC) will be chosen as the TapTalk algorithm.

### Sample size justification

The precise combination of blood-based biomarkers used to determine ‘positive’ preclinical AD pathology is yet to be determined as the scientific literature is developing quickly and new biomarkers are being developed regularly. We will review the literature before deciding on the best biomarker test, or combination of tests, that should be used to define AD pathology risk most accurately.

Currently p-tau 181 is considered one of the most accurate blood-based biomarkers for preclinical AD risk and we have therefore based our sample size calculation on this biomarker. As new tests are developed and are likely to be even more sensitive/specific than p-tau 181, the current calculation is a conservative estimate. We estimate that 17% of cognitively normal adults within our cohort have p-tau 181 in the preclinical AD range [55]. There are no tests of DDK compared to AD biomarkers, so we have based all calculations on finger tapping data.

We have performed ROC curve analysis using open data from Mollica et al. to calculate sensitivity and specificity of finger tapping speed and variability to predict CSF p-tau 181 [20]. This showed area under ROC curve (AUC) of 0.75 for a null linear model including age, gender and years of education, but not finger tapping. We calculated that the sample size to compare a screening test with an AUC > 0.90 against the null model requires 60 preclinical AD cases (positive p-tau181) and 290 healthy ageing controls (negative p-tau181). Thus, we will use 350 in the development dataset (expected 60 [17%] preclinical AD cases) and 150 in the validation dataset (expected 25 [17%] preclinical AD cases) to test cut-offs from the development model. The PROBAST tool [56] for assessment of prediction and diagnostic method studies confirms these development and validation strategies have a low risk of bias.

### AIM 2

#### Design

**Fig. 4:**
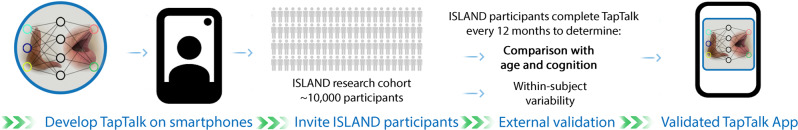
Development of smartphone application and assessment of usability and validity

There will be three sub-studies to address Aim 2 as follows: Study 2.1, a cross-sectional study of 30–40 adults to determine validity of data collection from different types of smartphones; Study 2.2, a prospective cohort study of 50–100 adults ≥ 50 years old to assess usability and reliability, and Study 2.3, a prospective cohort study of ∼1,000 adults ≥ 50 years old to assess validity against cognitive measures. The methods to addresses Aim 2 are summarised in Fig. 4. The TapTalk test protocol on a smartphone will be based upon the findings in Study 1 and comprise around three to five of the most discriminating hand and speech-like movements, aiming for test duration of approximately 2–3 min. The TapTalk protocol may be further refined after studies 2.1 and 2.2, depending on user feedback and results.

#### Participants and settings

##### Study 2.1

We will undertake a cross-sectional validation study of 30–40 participants who will be recruited from staff and students at the University of Tasmania. They will be invited to attend the research centre and use their own mobile phone to video record a member of the research team (who acts as a ‘dummy’ participant) as they perform a series of finger tapping tests whilst wearing Polhemus movement sensors (as the ‘gold standard’ motor data benchmark). The Polhemus electromagnetic tracking system delivers six degrees of freedom including the position (i.e., X, Y and Z coordinates of a space) and orientation (i.e., yaw, pitch and roll), of each object with a 60 Hz sample frequency. Compared to commonly used camera-based tracking systems, the Polhemus technology allow us to collect continuous and non-interrupted data without the issue of light-of-sight occlusions. This technology has been increasingly used in research on quantification of wrist and hand movements [57, 58]. Similarly, the participants will be asked to use their own smartphone microphone to record the speech-like sounds when the ‘dummy’ participant performs the DDK tasks. The audio benchmark for this will be data collected from a high-quality microphone (such as the Blue Yeti USB microphone). For each recording, the distance from the recording device (smartphone and microphone) and the dummy participant will be constrained to about 50 cm to allow for fair comparisons between the devices.

#### Statistical analysis

We will assess the accuracy of the video and audio data collected from each type of smartphone in comparison to the data collected by the wearable sensor measurements and high-quality microphone respectively, using correlation coefficients and mixed effects regression.

The results from this ‘smartphone validation’ study will: (i) allow a correction factor to be introduced, if necessary, into the data analysis later on for a wide range of smartphones and/or, (ii) provide assurance that the data collected for TapTalk on smartphones have little variation across a range of smartphones and in comparison to the movement and audio data collected by the gold -standard wearable sensors and microphone respectively.

##### Study 2.2

We will undertake a prospective study of 50–100 (randomly selected) ISLAND Project participants who participated in Study 1, own a smartphone, and live within 30 km of the University of Tasmania research centre. They will be invited to attend the University of Tasmania Clinical Research Facility in Hobart for approximately 15 min and asked to bring their own smartphone. Participants will be asked to install the TapTalk app on their own smartphone and complete a consent form. Each participant will then complete the test protocol of hand and speech-like movements in front of the researcher. This will allow the researcher to observe if there are any issues around the usability of installing and operating the TapTalk app.

Each participant will be asked to complete a User Experience Questionnaire immediately after completing both tests, to further evaluate the usability of each form of the test (see supplementary files). We will invite participants to complete TapTalk on a smartphone at least three times at home over the next 7-day period, using the same device each time. They will be sent a reminder email every day over the 7-day period and asked to complete the TapTalk once each day if possible, or at least a minimum of 3 times in total. Their performance on each test will be automatically uploaded to a central secure TapTalk database.

#### Statistical analysis

The responses to the User Experience Questionnaire, assessing usability, will be analysed with summary statistics. We will examine any practice effects (temporal reliability) by checking for increasing correlation between the first test (in the research centre) and repeated tests (at home) using Kendall rank correlation coefficients. A Kendall rank correlation coefficient of ≥ 0.70 is considered strong; 0.50 to 0.69 as good; 0.30 to 0.49 as moderate and < 0.30 as poor. This will be tested separately for each participant’s device.

The outcome of study 2.2 will be a measure of the usability and reliability of TapTalk. Based on the results from this pilot study, we will make refinements to the software and/or instructions and then invite all ISLAND participants to complete the TapTalk at home as part of study 2.3.

##### Study 2.3

We will undertake an 18-month prospective study of approximately 1,000 ISLAND Project participants who have completed online cognitive CANTAB tests in 2021. Participants in the ISLAND Project [45, 46] are invited to complete online CANTAB cognitive tests every 2 years. The first testing period was mid-2021 and ∼3,000 participants completed the tests; these participants will be invited to take part in the TapTalk prospective validity study. Every 6 months over an 18-month period (four data collection points in total; T0, T + 6, T + 12, T + 18), eligible participants will be invited via the ISLAND Project website (‘portal’), to complete TapTalk at any time over the next one-month period. Participants will be asked to complete TapTalk on their own smartphone at home or in a setting of their choice. Consent will be taken at each data collection point. When participants complete the TapTalk app they will be asked to complete a questionnaire about user experience and symptoms relating to hand and speech function (see supplementary files). The whole procedure, including the questions and tests, is expected to take about 5 min. A reminder email will be sent 1, 2 and 3 weeks after the initial invitation to people who have not yet completed the test.

#### Statistical analysis

The data from participants who complete TapTalk on two or more occasions over an 18-month period, and CANTAB on two occasions (2021 and 2023) will be included in the analysis. De-identified ISLAND Project participants will be classified as cognitively ‘stable’ or ‘declining’ using longitudinal CANTAB cognitive scores. TapTalk scores will be calculated using algorithms developed in Study 1. Logistic regression will be used to estimate the odds of a participant being confirmed as ‘declining’ at time T_2_ (24 months) conditioned on TapTalk score at time T_1_ (12 months). The overall outcome of Study 2 will be a smartphone app, TapTalk, that is usable, reliable and validated against established online CANTAB cognitive tests.

### Sample size justification

Using CANTAB cognitive test responses already collected in the ISLAND Project (n = 3,000), we estimate that we will collect > 1,000 complete cases (TapTalk app completion twice in 18 months and CANTAB at baseline and 24 months), which is ample for logistic regression with a single fixed-effect predictor variable.

### AIM 3

#### Design

**Fig. 5:**
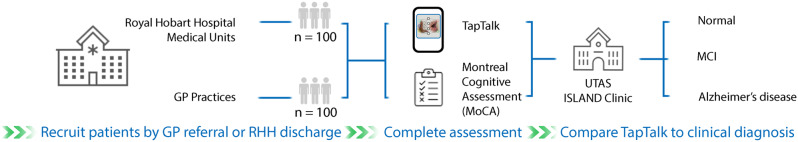
Prospective evaluation of usability and validity of TapTalk smartphone application (n = 200)

We will undertake a prospective study of 200 adults who *have cognitive symptoms* to validate TapTalk against gold-standard clinical consensus diagnosis of MCI and AD dementia. Secondary aims include determining the usability of TapTalk in a cohort with cognitive symptoms and comparing the usability and validity of TapTalk with commonly used brief screening tools. The study will be undertaken at two sites – the Royal Hobart Hospital (RHH) and the University of Tasmania ISLAND Cognitive Clinic with 100 people recruited from each site; see Fig. 5 for an overview of the study design.

The *inclusion criteria* are adults aged ≥ 50 years old with at least 3 months of persistent cognitive symptoms (patient- or family-reported).

The *exclusion criteria* are any adults who are < 50 years old, acutely unwell, have significant impairment of hand function or speech function, lack capacity to consent to the research study, or already have a known diagnosis of MCI or dementia.

### Recruitment from the hospital

There are several steps to recruiting eligible participants to the study and these are necessary as we will recruit from a pool of patients who are moving through their medical care at the RHH. Clinicians working at RHH (JA, NF, AB) will oversee the identification of 100 patients from the acute medical/subacute sites who may be eligible for the study. We will assess capacity to consent in line with standards of Good Clinic Practice research governance. Specifically, we will check that the participant has understood the information provided on the study, that they can retain it and can recount the key parts back to the researcher, that they can weigh up the information and that they can freely decide to participate.

After obtaining written consent, a research assistant (RA) will collect demographic and clinical details on the Data Collection Form (see supplementary files). The RA will ask the participant to complete the TapTalk assessment using the study smartphone; participants will be invited to hold the phone themselves, or to prop it up on a table, and to complete the TapTalk assessments without the RA prompting them (as it is designed as a ‘self-test’). The duration of testing is expected to take about 2–3 min. If the participant prefers the RA to help them, the RA will hold the smartphone and/or help guide participants through the tests as requested. They will make a note in the Data Collection Form (see supplementary files) how much help was administered and the indications for this, as this is valuable information for the research team to evaluate when considering usability of TapTalk in clinical cohorts.

The RA will then administer the MoCA, a standardised brief cognitive screening test that is commonly used in clinical practice and typically takes about 10–15 min to complete [59, 60]. The addition of this established cognitive test will allow for validation of the TapTalk scores, as well as comparison of the two tests (established cognitive test vs. TapTalk) compared to the clinical diagnosis (see later, ISLAND Clinic consensus diagnosis section). The MoCA outperforms the long-established MMSE in screening for cognitive impairment [60]. However, recent data suggest cultural differences may affect performance. Should the MoCA score suggest cognitive impairment for participants from culturally and linguistically diverse backgrounds or First Nations people, additional screening will be completed using the Rowland Universal Dementia Assessment Scale (RUDAS) [61].

After the participant has completed TapTalk and the cognitive test(s), the RA will ask the participant to complete a self-report ‘User Experience Questionnaire’ (see supplementary files). If the participant prefers the RA to read the questions out to them, the RA will do so and make a note of this in the Data Collection Form. This will provide valuable information on usability of TapTalk in a clinical cohort in comparison to a standard cognitive test.

After completing the test protocol, the RA will give the participant an information leaflet on the ISLAND Clinic (https://islandclinic.utas.edu.au/) that they can pass on to their GP if they would like to have further cognitive assessments. This GP letter will include a numerical research code used during the TapTalk assessments so that, if the participant attends the clinic, their data can easily be linked. The ISLAND Clinic protocol has been described previously in Alty et al. [62]; in brief it is a state-wide cognitive clinic that provides facilities and expertise for clinical diagnosis in a ‘one-stop’ interdisciplinary model where participants will have cognitive, medical and movement assessments performed on one day. They will also have an MRI brain scan, blood tests and an array of other tests. The interdisciplinary team formulates a consensus diagnosis for each participant attending the clinic. One of the clinicians on the team delivers the diagnosis to the participant on the same day and provides a detailed management plan for the participant and GP. The Clinic is supported by research funding and is Medicare bulk-billed for participants with no out-of-pocket costs.

This means that through the standard ISLAND Clinic processes, all participants recruited through the RHH will have the opportunity to have thorough medical and cognitive assessments, including neuropsychological tests, an interdisciplinary consensus diagnosis and management plan for their cognitive symptoms [62]. The established diagnosis of ‘cognitively unimpaired’ (objectively ‘normal’ on ISLAND Clinic tests), ‘MCI’ and ‘AD dementia’ (AD and mixed AD/vascular) also allow us to validate TapTalk scores against the gold standard clinical diagnosis.

### Recruitment from GP referrals via the ISLAND Cognitive Clinic

All patients aged ≥ 50 years who are referred to the ISLAND Clinic by their GP with at least 3 months of cognitive symptoms, and no established diagnosis of MCI or dementia, will be invited to take part in the TapTalk study. When participants attend the ISLAND Clinic, they are routinely assessed for their capacity to consent to research as part of ethics approved ISLAND Clinic procedures [62]. A researcher will administer the TapTalk and MoCA (or RUDAS) in the same way as described for participants recruited via the RHH. The only difference is that the clinical and demographic details described on the Data Collection Form are collected routinely within the ISLAND Clinic so will be cross populated from other assessments. For all participants seen in the ISLAND Clinic who were originally recruited through the hospital, the TapTalk, MoCA (or RUDAS), demographic and clinical data will have already been collected. The main purpose of the Clinic for these participants is to provide consent to access data collected during the Clinic, clarity on the diagnosis and a management plan for their cognitive symptoms. From the aspects of the TapTalk study, this diagnosis also allows us to validate the smartphone tests against clinical diagnosis.

### Statistical analysis

We will calculate AUC for TapTalk and MoCA (or RUDAS) to predict clinical diagnosis of MCI and AD dementia (defined as AD or mixed AD/vascular); 95% confidence intervals will be obtained using bootstrapping. Covariates may include age, gender, APOE4, years of education, and handedness. We will estimate cut-off scores for TapTalk and MoCA (or RUDAS) to differentiate between cognitively unimpaired vs. MCI, and between cognitively unimpaired vs. AD using the Youden index to optimise the trade-off between sensitivity and specificity. Classification accuracy (sensitivity and specificity) using these cut-offs will be compared using McNemar’s test. Participants diagnosed with other types of dementia (such as Dementia with Lewy Bodies or Frontotemporal Dementia) will not be included in this analysis.

### Sample size justification

As the conditional dependence between TapTalk and MoCA (or RUDAS) is unknown, the nominal power of the study cannot be calculated. However, Roalf et al. compared the diagnostic accuracy of MoCA with MMSE using a comparable sample size (n = 126 MCI and n = 140 healthy controls) and found a significant difference (in favour of MoCA) between the two diagnostic tests [63].

The main outcome of Study 3 will be an externally validated TapTalk smartphone test with determined precision for prospectively predicting risk of MCI and AD in adults ≥ 50 years old with cognitive symptoms. Secondly, we will have a measure of the accuracy and usability of the TapTalk smartphone test compared to MoCA in adults ≥ 50 years old with cognitive symptoms.

### Data management

All data obtained will be managed via a secure database which is hosted on the University of Tasmania virtual server managed by central Information Technology staff and backed up daily. Server access is restricted to authorised administrators using Secure Shell and Public Key Infrastructure certificates. Direct access to the databases is limited to system administrators and overseen by designated custodians of the data and will enable access to data in a de-identified fashion to research personnel. Data will be maintained in secure University of Tasmania databases for at least 10 years, and/or until 5 years after the final publication relating to this data. Consent will be sought for this long-term storage as well as linkage to extension projects. Specifically, data linkages with the ISLAND Project [45], TAS Test Project [38], and the ISLAND Clinic [62], are intended. Access will be requested through the University of Tasmania and the principal investigators of the studies. The participants in the ISLAND Project, TAS Test Project and the ISLAND Clinic all sign a consent form stating that they agree to their data being used for other unspecified research.

We will request that consent is provided to enable sharing of non-identifiable data with research collaborators external to the University of Tasmania. An open database of video recordings of hands/forearms, and audio recordings of DDK tasks will be developed as a resource for other researchers internal and external to University of Tasmania. Extended consent for future research participation will also be included in the consenting information. This is due to the potential opportunities for long-term follow-up in a specific cohort of participants with high risk of AD pathology. The study sponsor organisation is the University of Tasmania, Hobart, Australia, 7001. The study management group comprising clinicians, neuroscientists, computer scientists, and a statistician, will meet every 2 months to monitor and discuss the progress of the study, and to address any issues that may arise. Protocol deviations will be reported to the Human Research Ethics Committee in line with local recommendations.

If any unexpected adverse events (an unforeseen harmful, unpleasant or undesirable response, reaction or outcome experienced by a research participant or researcher) or serious adverse effects occur, these will be documented, reported in line with Good Clinical Practice, and appropriate referrals made for care. A participant may be discontinued from the study at any time if the participant or the research team feels that it is not in the participant’s best interest to continue. Possible reasons for discontinuation include participant withdrawal of consent, lost to follow-up, or new co-morbidity/diagnosis that would meet the exclusion criteria (e.g., loss of hand function). If the participant has taken part in completing TapTalk, and then changes their mind after data collection, the research team will do their best to withdraw all data from the study but if their de-identified data has already been included in the group analysis. it will not be possible to withdraw this data. Reasonable attempts will be made by the research team to provide a reason for participant withdrawals in data collection.

## Discussion

The planned outcome of the project is a new inexpensive smartphone test, TapTalk, to estimate the risk of preclinical AD, cognitive decline and AD dementia. If validated, this new scalable tool holds strong potential to transform dementia prevention and research globally. TapTalk will enable risk stratification of older adults and facilitate targeted interventions. The significant advantages of using a hand –and speech-like movement protocol are sensitivity to early preclinical AD and a protocol that has minimal language, educational or cultural barriers. The advantages of using an online test with standard smartphone equipment is the global reach of the internet crossing geographical barriers and providing accessibility for people in rural and remote communities and those in low-income countries.

Wide use of the new test would have significant societal, health, and economic impacts. The ability to non-invasively detect the risk of AD pathology would enable people with early-stage AD to be proactive *before* cognitive decline - to commence intense risk modification (that can slow/prevent 40% dementia) and potentially to enter drug trials. This would have direct benefits on dementia incidence and indirect benefits on other chronic diseases such as cardiovascular disease and cancer that share similar determinants, e.g. smoking, hypertension. It will also facilitate earlier diagnosis of dementia, which reduces hospital admissions and costs. There is evidence that less than half the number of people with dementia are actually diagnosed [64]. Those without a diagnosis have higher morbidity, three times more hospital admissions and three times higher healthcare costs [64, 65].Together, a test that aids detection of AD, supports clinical triage and diagnosis, and facilitates early intervention will help stem rising dementia care costs that are already >$15 billion/year in Australia [64]. Using our national and international networks for upscaling, such as the Australian Dementia Network (ADNeT) of clinics [66], the outcomes of this project have high potential to transform dementia prevention globally.

We have already recruited 1382 participants with a wide age range of 50–92 years, and a large amount of hand and speech-like movement data from TapTalk will be obtained through this study. This new database, probably the only one of its kind in the world, will be stored for future research use by University of Tasmania researchers and, with permission, for external researchers.

Potential risks of the study are acknowledged and strategies to mitigate these are discussed. There is a risk of inadequate recruitment, but we will mitigate this by recruiting existing participants in established research cohorts, namely the longitudinal cohorts of ISLAND Project and from the ISLAND Clinic where participants are primed to take part in research. There is a risk that TapTalk is not sufficiently accurate, but we have mitigated this by selecting component test items based on evidence of sensitivity to preclinical AD, combining multiple tests to amplify the multivariable model input data [> 10,000 data points], and electing well-established, transparent statistical modelling approaches that reveal the most discriminatory components of motor data, allowing further refinement.

We have devised a study protocol that plans to collect a sufficiently large dataset to employ multiple modelling methods, including feature-agnostic deep learning. A further risk is that participants lack a computer camera or smartphone to provide video-recorded hand movement data and audio speech-like data, or do not wish to do so; we have mitigated this risk by recruiting participants from studies that have online assessments already, offering the opportunity for participants to attend the clinical research centre in person if preferred, and providing two methods (video recorded and audio recorded data collection) within TapTalk.

It is conceivable that some participants will not want to know their dementia risk and this will hinder selection of participants for the clinical subset assessments. We have minimised this risk by recruiting from a study (ISLAND Project) [45] where participants understand the focus is on reducing dementia risk. It is also important to acknowledge that, as the bulk of this project relies on self-report of known neurological diagnoses, we have limited ability to make distinctions between other neurodegenerative disorders, including other forms of dementia and disorders which are correlated with dementia (e.g., Parkinson’s disease). Finally, there are risks around COVID-19 restrictions limiting recruitment or progress of the study; as most of the study is based around online movement and cognitive tests that can be completed at home, there are likely to be minimal effects and we have already collected the blood samples for p-tau 181 (and other biomarker) analyses.

In summary, this study directly addresses the critical need for population-level screening tests to detect the earliest stage of dementia. We take a completely new approach to developing a screening test to estimate the risk of AD pathology - through movement analysis, combining two simple hand- and oral-movement tests together that are sensitive across the continuum. Using movement analysis as the basis for an AD screening test has major advantages compared to cognitive tests in terms of crossing language, cultural and educational barriers; using a smartphone will overcome the barriers of other current AD biomarkers in terms of low cost, accessibility and non-invasiveness. There is thus high potential that TapTalk will provide a scalable screening approach – especially as the test equipment is already in our pockets.

### Electronic supplementary material

Below is the link to the electronic supplementary material.

Supplementary Material 1

Supplementary Material 2

Supplementary Material 3

Supplementary Material 4

Supplementary Material 5

## Data Availability

The datasets used and/or analysed during the current study will be available from the corresponding author on reasonable request. An ethics amendment will be required for additional sites to be added into the TapTalk project and research collaborations are welcomed.

## Abbreviations

Aβ: Amyloid beta
AD: Alzheimer’s disease
ADNeT: Australian Dementia Network
AI: Artificial intelligence
APOE: Apolipoprotein E: AUC: area under the curve
CANTAB: Cambridge Neuropsychological Test Automated Battery
COVID-19: Coronavirus disease of 2019
CSF: Cerebrospinal fluid
DDK: Diadochokinesis
FT: Finger tapping
GFAP: Glial fibrillary acidic protein
GP: General Practitioner
HREC: Human Research Ethics Committee
ISLAND: Island Study Linking Ageing and Neurodegenerative Disease
MCI: Mild Cognitive Impairment
MRI: Magnetic resonance imaging
NFL: Neurofilament light
NHMRC: National Health and Medical Research Council
PAL: Paired Associates Learning
PET: Positron emission tomography
p-tau: Phosphorylated tau
RA: Research assistant
ROC: Receiver Operating Characteristic
RHH: Royal Hobart Hospital
SD: Standard deviation
Simoa: Single molecule array
TAS Test: Tasmanian Test
UTAS: University of Tasmania
