# Supplementary material for: Development of a smartphone screening test for preclinical Alzheimer’s disease and validation across the dementia continuum
Source: BMC Neurol. 2024 Apr 16;24:127. doi: 10.1186/s12883-024-03609-z (PMC11020184; doi:10.1186/s12883-024-03609-z)
Supplement: Supplementary file 1 — Supplementary Material 1 [file 12883_2024_3609_MOESM1_ESM.docx]

**Supplementary file 1:** User questionnaire for assessing usability of TapTalk for use in Study 2.2 – in person pilot study at UTAS.

**Supplementary file 2:** Questionnaire on user experience and symptoms for Study 2.3, Prospective study – completed by participants at home online.

**Supplementary file 3:** TapTalk project data collection form for use in Study 3

**Supplementary file 4:** User experience questionnaire for use in Royal Hobart Hospital and The ISLAND Clinic in Study 3
