# Supplementary material for: Development of a smartphone screening test for preclinical Alzheimer’s disease and validation across the dementia continuum
Source: BMC Neurol. 2024 Apr 16;24:127. doi: 10.1186/s12883-024-03609-z (PMC11020184; doi:10.1186/s12883-024-03609-z)
Supplement: Supplementary file 2 — Supplementary Material 2 [file 12883_2024_3609_MOESM2_ESM.docx]

<For use in Study 2.2 – in person pilot study at UTAS>

**User Experience Questionnaire for assessing usability of TapTalk**

Thank you for taking part in the TapTalk research study. We would like to know what you think of the test so we can improve it further. Please complete the short questionnaire. It will take less than 5 minutes to complete.

1. **Which device do you usually perform ISLAND Project tests?**

- Smart phone
- Tablet
- Laptop
- Desktop
- Prefer not to say

**Questions 2-7: Please rate the following statements on a scale of 1 to 7** where 1 is “I strongly disagree” and 7 is “I strongly Agree”

1. **TapTalk looks attractive and friendly.**

| Strongly Disagree | Disagree | Moderately Disagree | Neural | Moderately Agree | Agree | Strongly Agree |
| --- | --- | --- | --- | --- | --- | --- |
| 1 | 2 | 3 | 4 | 5 | 6 | 7 |

1. **TapTalk was quick to perform.**

| Strongly Disagree | Disagree | Moderately Disagree | Neural | Moderately Agree | Agree | Strongly Agree |
| --- | --- | --- | --- | --- | --- | --- |
| 1 | 2 | 3 | 4 | 5 | 6 | 7 |

1. **TapTalk was easy to understand.**

| Strongly Disagree | Disagree | Moderately Disagree | Neural | Moderately Agree | Agree | Strongly Agree |
| --- | --- | --- | --- | --- | --- | --- |
| 1 | 2 | 3 | 4 | 5 | 6 | 7 |

1. **TapTalk seemed dependable and reliable.**

| Strongly Disagree | Disagree | Moderately Disagree | Neural | Moderately Agree | Agree | Strongly Agree |
| --- | --- | --- | --- | --- | --- | --- |
| 1 | 2 | 3 | 4 | 5 | 6 | 7 |

1. **I was motivated to perform well on the test.**

| Strongly Disagree | Disagree | Moderately Disagree | Neural | Moderately Agree | Agree | Strongly Agree |
| --- | --- | --- | --- | --- | --- | --- |
| 1 | 2 | 3 | 4 | 5 | 6 | 7 |

1. **If asked in the future, I would be happy to perform the test again.**

| Strongly Disagree | Disagree | Moderately Disagree | Neural | Moderately Agree | Agree | Strongly Agree |
| --- | --- | --- | --- | --- | --- | --- |
| 1 | 2 | 3 | 4 | 5 | 6 | 7 |

1. **Do you have suggestions to improve TapTalk?**

Please leave a comment.

**SUBMIT RESPONSES**

**This is the end of the questionnaire**

**Thank you for your time - your responses will help us develop this test.**
