# Supplementary material for: Development of a smartphone screening test for preclinical Alzheimer’s disease and validation across the dementia continuum
Source: BMC Neurol. 2024 Apr 16;24:127. doi: 10.1186/s12883-024-03609-z (PMC11020184; doi:10.1186/s12883-024-03609-z)
Supplement: Supplementary file 3 — Supplementary Material 3 [file 12883_2024_3609_MOESM3_ESM.docx]

<For Study 2.3, Prospective study –completed by participants at home online>

**Questionnaire on User Experience and Symptoms**

Thank you for taking part in the TapTalk research study. We would like to know what you think of the test so we can improve it further. We would also like to know about relevant symptoms affecting your hands and speech so this can be taken into account when analysing your data. Please complete the short questionnaire. It will take about 5 minutes to complete.

**1. Which device do you usually perform ISLAND Project tests?**

- Smart phone
- Tablet
- Laptop
- Desktop
- Prefer not to say

**2. Which device did you do TapTalk on** **today**?

- Smart phone
- Laptop

**Questions 3-8: Please rate the following statements on a scale of 1 to 7 where 1 is “I strongly disagree” and 7 is “I strong Agree”**

**3. TapTalk looks attractive and friendly.**

| Device | Strongly Disagree | Disagree | Moderately Disagree | Neural | Moderately Agree | Agree | Strongly Agree |
| --- | --- | --- | --- | --- | --- | --- | --- |
|  | 1 | 2 | 3 | 4 | 5 | 6 | 7 |

4.  **TapTalk was quick to perform.**

| System | Strongly Disagree | Disagree | Moderately Disagree | Neural | Moderately Agree | Agree | Strongly Agree |
| --- | --- | --- | --- | --- | --- | --- | --- |
|  | 1 | 2 | 3 | 4 | 5 | 6 | 7 |

**5. TapTalk was easy to understand.**

| System | Strongly Disagree | Disagree | Moderately Disagree | Neural | Moderately Agree | Agree | Strongly Agree |
| --- | --- | --- | --- | --- | --- | --- | --- |
|  | 1 | 2 | 3 | 4 | 5 | 6 | 7 |

**6. TapTalk seemed dependable and reliable.**

| System | Strongly Disagree | Disagree | Moderately Disagree | Neural | Moderately Agree | Agree | Strongly Agree |
| --- | --- | --- | --- | --- | --- | --- | --- |
|  | 1 | 2 | 3 | 4 | 5 | 6 | 7 |

**7. I was motivated to perform well on the test.**

| System | Strongly Disagree | Disagree | Moderately Disagree | Neural | Moderately Agree | Agree | Strongly Agree |
| --- | --- | --- | --- | --- | --- | --- | --- |
|  | 1 | 2 | 3 | 4 | 5 | 6 | 7 |

**8. If asked in the future, I would be happy to perform the test again.**

| System | Strongly Disagree | Disagree | Moderately Disagree | Neural | Moderately Agree | Agree | Strongly Agree |
| --- | --- | --- | --- | --- | --- | --- | --- |
|  | 1 | 2 | 3 | 4 | 5 | 6 | 7 |

**9. Do you have suggestions to improve TapTalk?**

Please leave a comment.

**Participant symptoms**

Your performance on some sections of the smartphone test (TapTalk) may be affected by difficulties moving your hands or mouth, pain in your hands or mouth, or feeling anxious. So these symptoms can be taken into account when we analyse your data, we would like to know about **how you are feeling today?**

| 1 | 2 | 3 | 4 | 5 | 6 | 7 | 8 | 9 | 10 |
| --- | --- | --- | --- | --- | --- | --- | --- | --- | --- |

**10. ‘Do you have any pain in your hands today?** Please indicate your level of pain on the scale of 1 to 10 below, where 10 is severe pain and 1 is no pain at all’.

| 1 | 2 | 3 | 4 | 5 | 6 | 7 | 8 | 9 | 10 |
| --- | --- | --- | --- | --- | --- | --- | --- | --- | --- |

**11. ‘Do you have any pain around your mouth today?** Please indicate your level of pain on the scale of 1 to 10 below, where 10 is severe pain and 1 is no pain at all’.

**12. ‘Do you feel any level of anxiety today?** Please indicate your level of anxiety on the scale of 1 to 10 below, where 10 is severe anxiety and 1 is no anxiety at all’.

| 1 | 2 | 3 | 4 | 5 | 6 | 7 | 8 | 9 | 10 |
| --- | --- | --- | --- | --- | --- | --- | --- | --- | --- |

**We would also like to know about any longer-term symptoms you have noticed over the past year so we can take these into account when analysing your TapTalk data:**

**13. Have you noticed any change in your WALKING over the past year?**

Yes ⃣ No ⃣

If no – skip to next question.

If yes, have you noticed any of these? (tick all that apply)

⃣ Slowing down

⃣ Speeding up

⃣ Falling over

⃣ Difficulty turning round

⃣ Worse posture

⃣ Other (free text) …………..…………………..…………………..…

**14. Have you noticed any change in your COGNITION (thinking, memory, concentration) over the past year?** Yes ⃣ No ⃣

If no – skip to next question;

If yes, have you noticed any of these? (tick all that apply)

⃣ Cannot remember dates

⃣ Cannot remember names

⃣ Word finding difficulties

⃣ Cannot concentrate properly

⃣ Lose things such as my keys and mobile phone

⃣ I get lost in unfamiliar places

⃣ I lose the thread of a conversation

⃣ Other (free text):…………………..…………………..…………………..…………………..…

**15. Have you noticed any change in THE WAY YOU SPEAK over the past year?**

Yes ⃣ No ⃣ If no – skip to next question; If yes, have you noticed any of these? (tick all that apply)

⃣ I speak more quietly

⃣ I speak louder

⃣ My speech is shaky/tremulous

⃣ My speech is slurred

⃣ My speech is slower

⃣ My speech is faster

⃣ Other (free text):……………..……………………..……………………..

**16. Is your speech affected by any of these? (tick all that apply)**

Stroke ⃣ Parkinson’s ⃣ Pain ⃣ Tremor/shaking ⃣ Dentures ⃣ Other ⃣
Details (free text):…………..…………………..…………………..……………………..

**17. Have you noticed any change in THE WAY YOUR HANDS WORK over the past year?**

Yes ⃣ No ⃣

If no – skip to next question; If yes, have you noticed any of these? (tick all that apply)

⃣ I struggle to open lids eg twisting the jam jar lid open

⃣ My writing has become smaller

⃣ My writing has become shaky

⃣ My writing has become bigger

⃣ I struggle to do fine motor tasks – eg doing up shirt buttons, tying my laces

⃣ I struggle to use tools – eg screwdriver, whisk

⃣ Other (free text):……………..……………………..……………………..

**18. Are your hands affected by any of these? (tick all that apply)**

Arthritis ⃣ Stroke ⃣ Parkinson’s ⃣ Pain ⃣ Other ⃣ Tremor/shaking ⃣
Details (free text):…………..…………………..…………………..……………………..

**SUBMIT RESPONSES**

**This is the end of the questionnaire**

**Thank you for your time - your responses will help us develop this test.**
