# Supplementary material for: Development of a smartphone screening test for preclinical Alzheimer’s disease and validation across the dementia continuum
Source: BMC Neurol. 2024 Apr 16;24:127. doi: 10.1186/s12883-024-03609-z (PMC11020184; doi:10.1186/s12883-024-03609-z)
Supplement: Supplementary file 4 — Supplementary Material 4 [file 12883_2024_3609_MOESM4_ESM.docx]

<To be completed by the Research Assistant who will administer the questions in person to the participant (data recorded in electronic or paper form) in Study 3>

**TapTalk Project Data Collection Form**

**Date of assessment: __________________**

**Place of assessment:** Hospital RHH/Repat/University of Tasmania

Ward/Outpatient clinic/UTAS Clinical Research Facility

**Participant details:**

- **What is your name?**
- **What is your date of birth?**
- **What is your gender?** male/female/other/prefer not to say
- **What hand do you write with?** Right/left/either
- **How many full times years of education did you complete?**
- **Can you read English OK?** If NO – consider using RUDAS rather than MOCA (and offer to read the instructions on the smartphone test out to the participant)

Note to RA: please allocate a research code for this participant in the ‘code key’ document and only use this code when starting the smartphone TapTalk

Do not enter the research code on this document

**Participant symptoms**

Your performance on some sections of the smartphone test (TapTalk) may be affected by difficulties moving your hands or mouth, pain in your hands or mouth, or feeling anxious. So these symptoms can be taken into account when we analyse your data, we would like to know about **how you are feeling today?**

| 1 | 2 | 3 | 4 | 5 | 6 | 7 | 8 | 9 | 10 |
| --- | --- | --- | --- | --- | --- | --- | --- | --- | --- |

**1. ‘Do you have any pain in your hands today?** Please indicate your level of pain on the scale of 1 to 10 below, where 10 is severe pain and 1 is no pain at all’.

| 1 | 2 | 3 | 4 | 5 | 6 | 7 | 8 | 9 | 10 |
| --- | --- | --- | --- | --- | --- | --- | --- | --- | --- |

**2. ‘Do you have any pain around your mouth today?** Please indicate your level of pain on the scale of 1 to 10 below, where 10 is severe pain and 1 is no pain at all’.

**3. ‘Do you feel any level of anxiety today?** Please indicate your level of anxiety on the scale of 1 to 10 below, where 10 is severe anxiety and 1 is no anxiety at all’.

| 1 | 2 | 3 | 4 | 5 | 6 | 7 | 8 | 9 | 10 |
| --- | --- | --- | --- | --- | --- | --- | --- | --- | --- |

**We would also like to know about any longer-term symptoms you have noticed over the past year so we can take these into account when analysing your TapTalk data:**

**4. Have you noticed any change in your WALKING over the past year?**

Yes ⃣ No ⃣

If no – skip to next question.

If yes, have you noticed any of these? (tick all that apply)

⃣ Slowing down

⃣ Speeding up

⃣ Falling over

⃣ Difficulty turning round

⃣ Worse posture

⃣ Other (free text) …………..…………………..…………………..…

**5. Have you noticed any change in your COGNITION (thinking, memory, concentration) over the past year?** Yes ⃣ No ⃣

If no – skip to next question;

If yes, have you noticed any of these? (tick all that apply)

⃣ Cannot remember dates

⃣ Cannot remember names

⃣ Word finding difficulties

⃣ Cannot concentrate properly

⃣ Lose things such as my keys and mobile phone

⃣ I get lost in unfamiliar places

⃣ I lose the thread of a conversation

⃣ Other (free text):…………………..…………………..…………………..…………………..…

**6. Have you noticed any change in THE WAY YOU SPEAK over the past year?**

Yes ⃣ No ⃣ If no – skip to next question; If yes, have you noticed any of these? (tick all that apply)

⃣ I speak more quietly

⃣ I speak louder

⃣ My speech is shaky/tremulous

⃣ My speech is slurred

⃣ My speech is slower

⃣ My speech is faster

⃣ Other (free text):……………..……………………..……………………..

**7. Is your speech affected by any of these? (tick all that apply)**

Stroke ⃣ Parkinson’s ⃣ Pain ⃣ Tremor/shaking ⃣ Dentures ⃣ Other ⃣
Details (free text):…………..…………………..…………………..……………………..

**8. Have you noticed any change in THE WAY YOUR HANDS WORK over the past year?**

Yes ⃣ No ⃣

If no – skip to next question; If yes, have you noticed any of these? (tick all that apply)

⃣ I struggle to open lids eg twisting the jam jar lid open

⃣ My writing has become smaller

⃣ My writing has become shaky

⃣ My writing has become bigger

⃣ I struggle to do fine motor tasks – eg doing up shirt buttons, tying my laces

⃣ I struggle to use tools – eg screwdriver, whisk

⃣ Other (free text):……………..……………………..……………………..

**9. Are your hands affected by any of these? (tick all that apply)**

Arthritis ⃣ Stroke ⃣ Parkinson’s ⃣ Pain ⃣ Other ⃣ Tremor/shaking ⃣
Details (free text):…………..…………………..…………………..……………………..

**10**. RA asks: ‘**Do you recall completing the Montreal Cognitive Assessment (also known as the MoCA) within the last few months?**’ and briefly show the participants MoCA version 1

YES – use MoCA alternate version

NO – use MoCA version 1

**11. Do you identify as Aboriginal or from the Torres Strait Islands?**

If yes, consider using RUDAS after completing the MoCA (if MoCA score is <26/30)

**RA states: ‘This is the end of the questionnaire.**

**Now I will show you how to do the smartphone test’**

**RA – please note down:**

**(a) which cognitive test was used**:

MOCA v 1 / MOCA alternate version/ RUDAS

**(b) How much help did you give to the participant when completing the:**

- **Hand tests?** none/ a little / a lot/ read all instructions out to them
- **The speech tests?** none/ a little / a lot/ read all instructions out to them

Details….(free text):
